# Tracheal Intubation with Aura-i and aScope-2: How to Minimize Apnea Time in an Unpredicted Difficult Airway

**DOI:** 10.1155/2015/453547

**Published:** 2015-01-06

**Authors:** Vittorio Pavoni, Valentina Froio, Alessandra Nella, Martina Simonelli, Lara Gianesello, Andrew Horton, Luca Malino, Massimo Micaglio

**Affiliations:** ^1^Department of Anesthesia and Intensive Care, University-Hospital Careggi, Largo Brambilla 3, 50134 Firenze, Italy; ^2^Faculty Practice Group, University of California, Los Angeles, CA 90095, USA; ^3^Ambu Srl, Via Paracelso 18, Agrate Brianza, 20041 Milano, Italy

## Abstract

The supraglottic airway's usefulness as a dedicated airway is the subject of continuing development. We report the case of an obese patient with unpredicted difficult airway management in which a new “continuous ventilation technique” was used with the Aura-i laryngeal mask and the aScope-2 devices. The aScope-2/Aura-i system implemented airway devices for the management of predictable/unpredictable difficult airway. The original technique required the disconnection of the mount catheter from Aura-i, the introduction of the aScope-2 into the laryngeal mask used as a conduit for video assisted intubation and then towards the trachea, followed by a railroading of the tracheal tube over the aScope-2. This variation in the technique guarantees mechanical ventilation during the entire procedure and could prevent the risk of hypoventilation and/or hypoxia.

## 1. Introduction

Supraglottic airway devices (SADs) play a critical role in the management of difficult airway and their use in patients with difficult face-mask ventilation or failed tracheal intubation is widely recommended [1, 2]. Furthermore, SADs are well described as conduits to facilitate tracheal intubation [3], although most devices require fiber-optic bronchoscope (FOB) guidance to increase the rate of success [4–6]. With the aim of minimizing apnea time during the entire procedure, improving patient's safety, a continuous ventilation technique during the intubation procedure has been described, using a mount catheter with a fiber-optic cap attached to the tracheal tube (TT) [7].

We report a case of unpredicted difficult airway in which fiber-optic intubation was performed using a new “continuous ventilation technique” with combination of the Aura-i disposable laryngeal mask (Ambu A/S, Ballerup, Denmark) and the aScope-2 (Ambu A/S, Ballerup, Denmark).

The precurved disposable laryngeal mask Aura-i (Ambu A/S, Ballerup, Denmark) is a SAD designed to facilitate one-step tracheal intubation with FOB guidance [8], due to its anatomically correct curve and the presence of a navigation mark for guiding flexible scope. Its role has become still more interesting since the development of the aScope-2 (Ambu A/S, Ballerup, Denmark), a flexible intubation scope with its high-resolution specific monitor. Both devices are single use in order to reduce cross contamination and cleaning or repair costs [9].

## 2. Case Report

**Figure 1:**
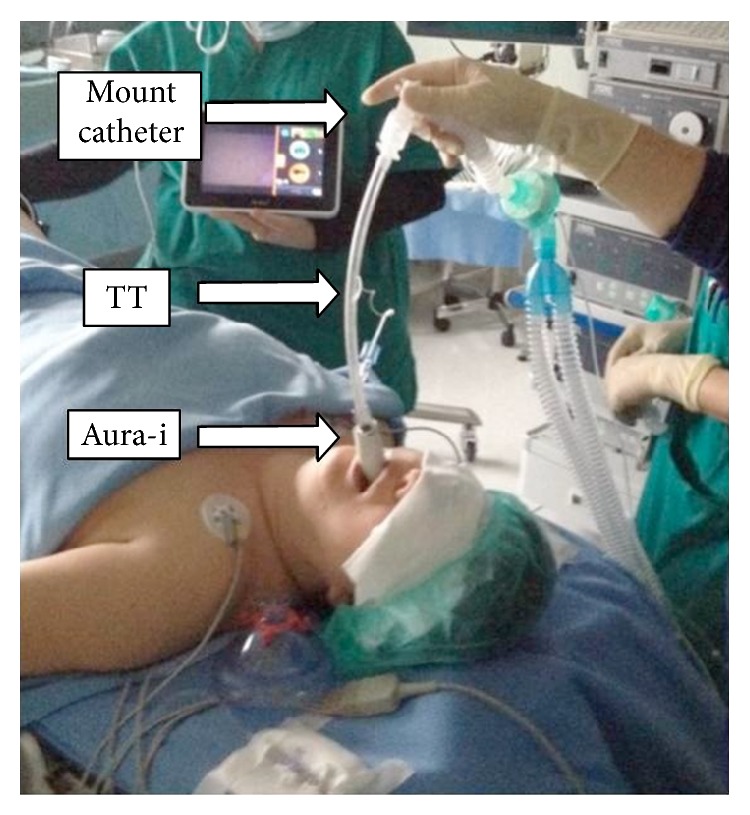
The patient is mechanically ventilated through a tracheal tube (TT) partially inserted in the airway tube of Aura-i and with the cuff inflated.

A 55-year-old female was scheduled to undergo elective cholecystectomy. Past medical history was significant for obesity (body mass index 31.2 Kg/m^2^, obesity class I), tobacco smoking, and mild chronic obstructive pulmonary disease, treated with short-acting *β*_2_-agonists. Preoperative arterial blood gas analysis showed PaO_2_ 66 mmHg, PaCO_2_ 42 mmHg, HCO_3^−^_ 29 mEq/L, pH 7.39, and BE −3.0. On preoperative airway examination the Mallampati score oral opening view was evaluated to be class 2, with mouth opening of approximately 4 cm, a thyromental distance of 6.5 cm, and jaw protrusion was estimated as grade B and we noted also slightly limited neck extension. The patient exhibited slightly limited craniocervical extension. A borderline situation for possible difficult intubation was identified, but except for a light increase of BMI, no predictor of difficult mask ventilation was found. After routine monitoring, preoxygenation was performed and anesthesia was induced with propofol 170 mg and fentanyl 150 mcg i.v. After confirmation of the ability to ventilate the patient's lungs using a bag and mask, 80 mg of rocuronium was administered. Direct laryngoscopy was then performed, which revealed a grade III laryngeal view (Cormack and Lehane) due to a large floppy epiglottis. One attempt of gum elastic bougie-assisted tracheal intubation technique was unsuccessful. A size 4 Aura-i laryngeal mask was then easily inserted and the cuff inflated up to a pressure of 50–60 cmH_2_O. Satisfactory ventilation was achieved in few seconds after connection to the ventilator (Drager Primus, Dragerwerk AG & Co.) using a volume controlled model (tidal volume 7 mL/kg, respiration rate 10 per min, PEEP 5 cmH_2_O) and we considered the use of the SAD to facilitate tracheal intubation. With the aim of preventing hypoxia during the procedure, reducing the time of apnea, a variation in the intubation technique suggested from the manufacturer was introduced. Without discontinuing mechanical ventilation, the aScope-2 was guided inside the airway tube of Aura-i through a mount catheter with a fiber-optic cap (DAR-Covidien) and both its position and its anatomical relation with the glottis were checked. A partially obstructed view at the level of distal orifice of the Ambu-I LM was noted during the initial view; this was probably caused by malposition or downfolding of epiglottis. Following “up and down movements” of the LM, the view cleared up, with full view of the glottis displayed on the monitor, and the aScope-2 was withdrawn from the mask. The mount catheter was disconnected from the ventilatory circuit for few seconds to allow the distal end of a well lubricated 7.5 mm ID TT to be partially inserted into the airway tube of Aura-i. The TT cuff was inflated and the tube was connected to ventilator with unchanged ventilation mode. Then TT cuff was deflated and the TT was advanced over aScope-2 that was placed in the trachea and the TT connector hooked up to the anaesthesia circuit (Figure 1). Without discontinuing ventilation, the 630 mm insertion-cord of the aScope-2 was first guided inside the TT through the fiber-optic cap of the mount catheter and then into the airway tube of Aura-i to gain a full view of the glottic opening from the end of the SAD.

The cuff of the tracheal tube was deflated and the tube was then advanced over the aScope-2 used as a guide. Connector of the tracheal tube was attached to the anaesthesia circuit (Figure 1). Correct depth of the tracheal tube was checked and the aScope-2 was subsequently withdrawn. The TT cuff was inflated while the Aura-i cuff was deflated and kept in place, decreasing pressure on the pharyngeal mucosa. Anaesthesia and surgery proceeded uneventfully.

## 3. Discussion

Hypoxia may complicate difficult airway management representing a life-threatening event [10]. Obese patients have an elevated risk of hypoxia as compared to the nonobese population. The use of a SAD has been recently recommended in obese population instead of the face-mask ventilation in order to ease airway management and administer PEEP, thus preventing hypoxia [11].

Our patient did not show any feature that suggested difficulty in face-mask ventilation; therefore the early placement of SAD appeared not essential. On the other hand, our patient showed difficulty on direct laryngoscopy with tracheal intubation which was not predicted on the base of preoperative airway evaluation, if we exclude Mallampati class 2 and jaw protrusion grade B.

The usefulness of SADs both in unpredicted difficult airway and in predicted difficult intubation with conventional laryngoscopy was the subject of considerable interest [12, 13]. A number of studies have reported intubation through a SAD using a blind technique or assisted by light wands, optical stylets, and flexible fiber optic [14, 15]. Individual case reports have also described a variety of techniques.

**Figure 2:**
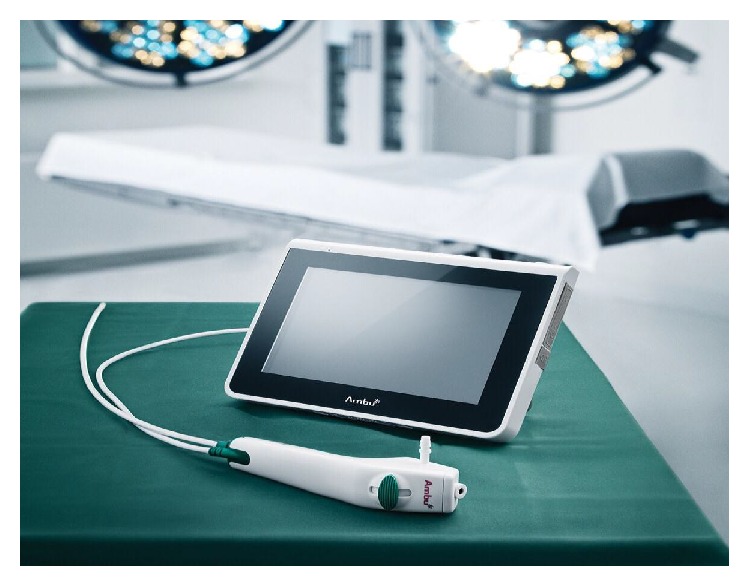
The aScope-2 with a transportable monitor.

The aScope-2/Aura-i system implements airway devices for the management of predictable/unpredictable difficult airway. The light weight of the “ergonomically designed” handle of the aScope-2 makes it easy to manipulate [16] (Figure 2). Furthermore the aScope-2 is less rigid than a fiberscope sparing the endotracheal tube to find resistance to progression through the laryngeal mask and the vocal cords. The portable monitor makes it easy to transport from one operating room to the other one and makes the system even more simple and friendly to use. Both the Aura-i laryngeal mask and the aScope-2 are disposable devices, so biological risks are minimized and they can be used in patients with infectious and transmissible diseases. They are always ready to use and there is no risk of damage during decontamination and storage.

The aScope-2 has a poor fiber-optic view comparing to a fiberscope and its tip is characterized by a lesser range of movement, with a limited angulation [9]. The aScope-2 has no suction port. Since outer diameter of the aScope-2 (5.3 mm) is larger than that of adult reusable fiber-optic scopes (3.5–4.2 mm), the Aintree Intubation Catheter, a device specifically designed to aid fibrescope guided tube placement through a laryngeal mask airway, cannot be used with it. When using a size 3 Aura-i laryngeal mask, a maximal ID size of the TT through this device is 6.5 mm. Positive pressure ventilation may be difficult through combination of the 6.5 mm ID TT and the aScope-2 [17]. The original technique needs the disconnection of the mount catheter from Aura-i, the introduction of the aScope-2 into the mask and then towards the trachea, followed by the introduction of the tracheal tube through the aScope-2. The variable time of apnea experienced during tracheal tube positioning could become critical in situations where the patient presents comorbidities that increase the risk related to hypoventilation and/or hypoxia. As previously described by Hammarskjöld et al. [18], conversion from laryngeal mask to endotracheal tube may be difficult. In their paper, where they intubated patients using a bougie guided technique through a laryngeal mask with a fiber-optic bronchoscope, the experienced time of apnoea was extremely variable: 17 patients were intubated within 2 minutes, 11 patients were intubated between 2 and 5 minutes, and one patient required 10 minutes. Variation of the intubation technique through the Aura-i laryngeal mask described in this case report guarantees adequate ventilation during the entire procedure and could prevent this risk.

## 4. Conclusion

Applying positive pressure ventilation during fiber-optic intubation through the SAD can reduce the potential risk of hypoxia and hypercapnia in case of unanticipated difficult airway.
